# Design and Investigation of Optical Properties of *N*-(Rhodamine-B)-Lactam-Ethylenediamine (RhB-EDA) Fluorescent Probe

**DOI:** 10.3390/s18041201

**Published:** 2018-04-14

**Authors:** Eva Soršak, Julija Volmajer Valh, Špela Korent Urek, Aleksandra Lobnik

**Affiliations:** 1 Institute of Engineering Materials and Design, Faculty of Mechanical Engineering, University of Maribor, Smetanova 17, 2000 Maribor, Slovenia; sorsak.eva@gmail.com (E.S.); julija.volmajer@um.si (J.V.V.); 2 Institute for Environmental Protection and Sensors, Beloruska 7, 2000 Maribor, Slovenia; spela@vips.si

**Keywords:** rhodamine B, ethylenediamine, chemical modification, fluorescence quenching, optical detection, Ag^+^ ions

## Abstract

This study presents chemical modification of a Rhodamine B (RhB) sensor probe by ethylenediamine (EDA), and investigation of its spectral as well as sensor properties to the various metals. The synthesised *N*-(Rhodamine-B)-lactam-ethylenediamine (RhB-EDA) fluorescent probe shows interesting optical sensor properties, and high sensitivity and selectivity to Ag^+^ ions among all the tested metal ions (K^+^, Mg^2+^, Cu^2+^, Ni^2+^, Fe^2+^, Pb^2+^, Na^+^, Mn^2+^, Li^+^, Al^3+^, Co^2+^, Hg^2+^, Sr^2+^, Ca^2+^, Ag^+^, Cd^2+^ and Zn^2+^), while the well-known Rhodamine B (RhB) fluorescent probe shows much less sensitivity to Ag^+^ ions, but high sensitivity to Fe^2+^ ions. The novel fluorescent sensor probe RhB-EDA has the capabilities to sense Ag^+^ ions up to µM ranges by using the fluorescence quenching approach. The probe displayed a dynamic response to Ag^+^ in the range of 0.43 × 10^−3^–10^−6^ M with a detection limit of 0.1 μM. The sensing system of an RhB-EDA novel fluorescent probe was optimised according to the spectral properties, effect of pH and buffer, photostability, incubation time, sensitivity, and selectivity. Since all the spectral and sensing properties were tested in green aqueous media, although many other similar sensor systems rely on organic solvent solutions, the RhB-EDA sensing probe may be a good candidate for measuring Ag^+^ ions in real-life applications.

## 1. Introduction

Silver is one of the most poisonous ions that can be found throughout the ecosystems. It is believed that the toxicity of silver originates mainly from the ability of silver ion (Ag^+^) to bind with various metabolites and sulfhydryl enzymes, leading to the inactivation of enzymes and, consequently, to the resulting health problems [1]. In addition, it is assumed that the Ag^+^ ions implement adverse effects in patients who overuse medication containing silver salts, causing accumulation in liver tissue [2].

People are exposed to silver due to its wide use in industrial products, such as photographic imaging agents and pharmaceutical products, and consumer products, such as toothpastes, soaps, and deodorants as antimicrobial agents. Human activities, such as alkali and metal processing, incineration of coal, water, and soil, therefore, increase the likelihood of exposure of people to such highly toxic metal. The US Environmental Protection Agency (EPA) has defined a Secondary Maximum Contaminant Level (SMCL) in drinking water for this metal at 0.1 mg L^−1^ [3]. Thus, the development of probes possessing high selectivity for Ag^+^ ions over other commonly coexistent metal ions in various media is of considerable significance for environmental protection and human health [2].

Although traditional techniques have been applied widely to detect Ag^+^ ions in environmental samples, such as gas chromatography and liquid chromatography [4], atomic absorption/emission spectroscopy [5], electro thermal atomic absorption spectrometry [6], Inductively Coupled Plasma-Mass Spectrometry (ICP-MS) [7] and Inductively Coupled Plasma-Atomic Emission Spectrometry (ICP-AES) [8], they require expensive and sophisticated instrumentation and/or time-consuming and complicated sample preparation processes [9]. Therefore, they are not well suited for rapid on-site detection of Ag^+^ ions. Many probe-based sensors intended for on-site detection using organic chromophores, small fluorescent organic molecules [10,11,12], Quantum Dots (QDs) [13,14], conjugated polymers [15], and DNA-zymes [16] have been developed for fluorescent, colorimetric [17,18,19], and electrochemical detection of Ag^+^ [20,21]. However, some drawbacks still exist, such as poor aqueous solubility [18,22], bad stability, low sensitivity, large physical size, complicated synthesis procedures, high toxicity, and cross-sensitivity toward other metal ions, especially to Hg^2+^ [22,23,24,25]. 

Herein, a probe based on fluorophore Rhodamine B (RhB) was synthesised [26]. It was previously used as an intermediate in the synthesis of various rhodamine derivates [26,27,28] and dendrimers [29,30]. However, to the best of our knowledge, its interesting optical properties and sensitivity to Ag^+^ ions were overlooked. The optical properties of RhB-EDA (**3**) and its sensitivity to Ag^+^ ions are presented in the proposed article. The sensing characteristics of an RhB-EDA probe were tested in green aqueous (90 *v*/*v* % H_2_O, 10 *v*/*v* % DMSO) media rather than in different water-organic solvent solutions. The results suggest that RhB-EDA shows great potential as a fluorescent probe for the detection of Ag^+^ ions in aqueous samples because of its simple synthesis and high selectivity toward Ag^+^ ions.

## 2. Materials and Methods

### 2.1. Chemicals and Starting Materials

The Rhodamine B, etilenediamine (EDA), methyl acrylate, methanol, dimethyl sulfoxide (DMSO), FeSO_4_·7H_2_O, CuSO_4_·5H_2_O, PbSO_4_, CoCl_2_·6H_2_O, Sr(NO_3_)_2_, CaCl_2_·2H_2_O, Zn(NO_3_)_2_, Ag_2_SO_4_, Hg(NO_3_)_2_·H_2_O, CdSO_4_·2.67H_2_O were from Sigma Aldrich (Slovenia). MgCl_2_·6H_2_O, NaNO_3_ and KNO_3_ were provided by Alkaloid Skopje. NiCl_2_·6H_2_O, LiCl, AlCl_3_·6H_2_O and Mn(CH_3_COO)_2_·6H_2_O were purchased from Kemika (Slovenia). The Na_2_CO_3_ and NaHCO_2_ were provided by Riedel-de Haën (Slovenia). All of the reagents were used as received. The solvent used in synthesis was of analytical grade.

### 2.2. Synthetic Procedure and Characterisation Data for RhB-EDA

RhB (**1**) (4 g, 8.35 mmol) was added to an excess of EDA (**2**). After refluxing for 2 h, the mixture was cooled to room temperature and quenched with water (100 mL). Then, the mixture was filtered. The crude product thus obtained was dried in a vacuum dryer at RT to give the compound RhB-EDA (**3**) as an amber white solid (3.68 g, 92% yield). m.p. 218–219 °C in lit 217–219 °C [28]; ^1^H-NMR (DMSO-d_6_, 500 MHz), *δ* 7.79–7.74 (*m*, 1H, ArH), 7.52–7.45 (*m*, 2H, ArH), 7.02–6.97 (*m*, 1H, ArH), 6.40–6.30 (*m*, 6H, ArH), 3.30–3.34 (*m*, 8H, NCH_2_CH_3_), 3.00–2.92 (*m*, 2H, NCH_2_CH_2_NH_2_), 2.18 (*dd*, *J* = 8.7, 6.5 Hz, 2H, NCH_2_CH_2_NH_2_), 1.08 (*t*, *J* = 7.0 Hz, 12H, NCH_2_CH_3_) (the exchangeable NH_2_ signal is hidden); ^13^C-NMR (125 MHz, CDCl_3_) *δ* 12.6, 40.8, 43.9, 44.3, 64.8, 97.6, 105.6, 108.1, 122.7, 123.8, 127.9, 128.7, 131.2, 132.3, 148.7, 153.2, 153.4, 168.5; IR (ATR, *ν*, cm^−1^): 669, 703, 764, 783, 812, 867, 923, 969, 1016, 1078, 1117, 1151, 1200, 1218, 1232, 1266, 1307, 1329, 1352, 1375, 1427, 1466, 1480, 1514, 1542, 1584, 1615, 1682, 2928, 2968.

### 2.3. Solutions

The RhB-EDA stock solution was prepared by dissolving in DMSO to form a 1 mM stock solution. Since RhB-EDA alone is not soluble in water, stock solution was prepared in a small amount of DMSO. RhB and metal salts were dissolved in distilled water to yield 0.1 µM and 1 mM stock solutions, respectively.

The MOPS (3-(*N*-morpholino)propanesulfonic acid) buffer was obtained by dissolving 2.093 g of 3-morpholinopropanesulfonic acid in approximately 900 mL of distilled water. Before being filled up to 100 mL, the solution was adjusted to pH 7 using 70% perchloric acid. To make the tris(hydroxymethyl)aminomethane (TRIS) buffer, we dissolved 2.092 g of bis-tris base in 900 mL of distilled water. Before being filled up to 1000 mL, the solution was adjusted to pH 7 using 70% perchloric acid. The hydroxyethyl-piperazineethane-sulfonic acid (HEPES) buffer was obtained by dissolving 2.380 g of 3-morpholinopropanesulfonic acid in approximately 900 mL of distilled water. Before being filled up to 1000 mL, the solution was adjusted to pH 7 using 70% perchloric acid. A Phosphate Buffer Stock (PBS) solution was prepared by mixing adequate quantities of 0.1 M KH_2_PO_4_ and 0.1 M NaOH in distilled water.

Working solutions were prepared using distilled water, buffer and RhB-EDA stock solution to obtain a final RhB-EDA concentration of 10^−4^ M. The stock solution of RhB-EDA and working solutions with different pH values were prepared daily. The test samples were incubated for 20 min at room temperature prior to measurements.

When the sensing behaviour of RhB-EDA toward metal ions was studied, the stock solutions of RhB-EDA were mixed with metal stock solution, buffer and water in a molar ratio 3:3:14:10. The final concentration of RhB-EDA and metal ions were 0.1 mM. Solutions were incubated for 20 min at room temperature prior to measurements.

### 2.4. Instrumentation

^1^H NMR spectra were recorded with a 500 MHz spectrometer and are reported in ppm using TMS (tetramethylsilane) as an internal standard. ^13^C NMR spectra were recorded by a 125 MHz spectrometer. The coupling constants (*J*) are given in Hz. Multiplicities are indicated as follows: s (singlet), d (doublet), t (triplet), q (quartet), sep (septet) or m (multiplet). The melting point was determined using a microscope hot stage, and was uncorrected. 

UV-VIS spectra were recorded on a PerkinElmer Lambda35 UV/VIS spectrophotometer (supplied by Omega d.o.o., Slovenia). The fluorescent data were taken on a PerkinElmer LS 55 luminescence spectrometer equipped with a Xenon discharge lamp. The excitation and emission slits were varied between 5 and 9 nm. ATR FT-IR spectra were recorded on a Perkin Elmer Spectrum GX spectrometer. The ATR accessory (supplied by Specac Ltd., Orpington, UK) contained a diamond crystal. A total of 16 scans for each sample were taken with a resolution of 4 cm^−1^. All spectra were recorded at 21 °C over the wavelength interval between 4000 and 650 cm^−1^. Mass spectra was performed on a Waters Micromass Q-Tof Premier (supplied by Waters Corporation, Vienna, Austria).

### 2.5. Fluorescence Quantum Yield

The procedure for the determination of RhB-EDA quantum yield was carried out as described elsewhere [31]. Rhodamine G was used as the standard. A quantum yield (*Φ_s_*) of RhB-EDA was estimated from the fluorescence spectra of RhB-EDA according to Equation (1), where the subscripts s and r stand for the sample and the reference (rhodamine G6, *Φ_r_* = 0.95 in ethanol), respectively. *Φ* is the quantum yield, *A* represents the absorbance at the excitation wavelength, *S* refers to the integrated emission band areas, and *n_d_* is the solvent refractive index:

(1) $$\Phi_{s}=\Phi_{r}\frac{S_{s}}{S_{r}}\frac{A_{r}}{A_{s}}\frac{n_{Ds}^{2}}{n_{Dr}^{2}}.$$

## 3. Results

### 3.1. Design and Synthesis of RhB-EDA

In the past, several Ag^+^ ions’ probes have been synthesised [11,12,15,22,32]. Nevertheless, these syntheses are multistep processes, and the production is expensive, as well as time consuming. Moreover, for the synthesis, organic solvents are often used, and synthesised probes are mostly tested in water-organic solutions. The long-term stability of those probes is also questionable [33]. Therefore, we wanted to synthesise a probe for Ag^+^ ions that would be stable, relatively inexpensive, easy to produce and useful in water samples.

**Scheme 1:**
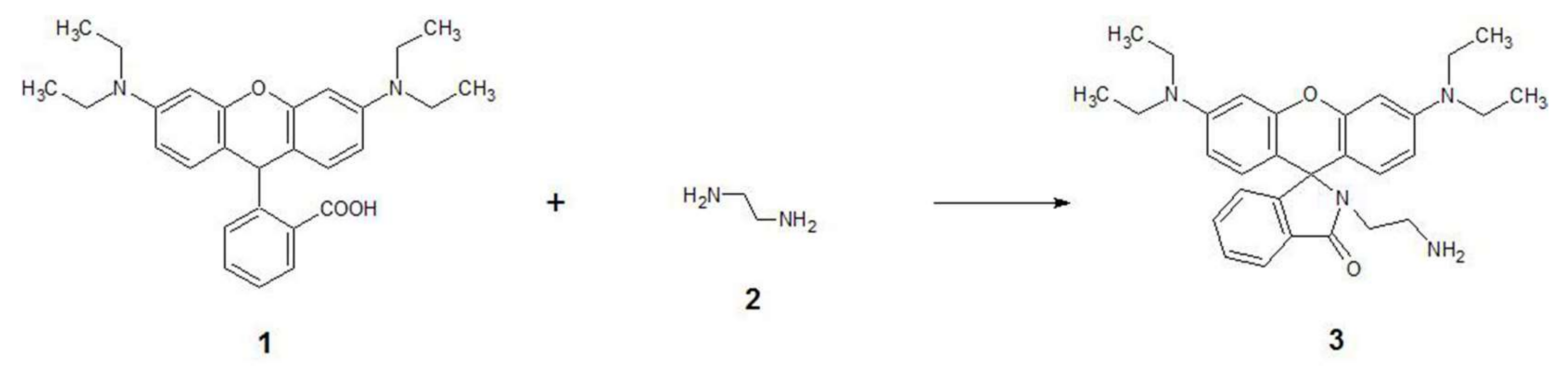
Synthesis of RhB-EDA.

RhB-EDA (**3**) was synthesised by treating RhB (**1**) with EDA (**2**), which was followed by precipitation by water. After filtering the crude product and its drying in a vacuum dryer, RhB-EDA (**3**) was obtained in a 93% yield (Scheme 1)

The probe was stored in the dark at Room Temperature (RT) and showed no changes in the structure, as well in the optical properties, after one year.

### 3.2. Spectral Properties of Synthesised RhB-EDA Fluorescent Probe

RhB is, in the form of green crystals or reddish-violet powder, mixed with water to make a bright pink colour. RhB-EDA is a yellow-white powder that has rather different optical properties. RhB alone shows optical properties with λ_ex_ and λ_em_ at 555/575 nm, while RhB-EDA shows λ_ex_ and λ_em_ at 500/538 nm; RhB-EDA shows negative solvatochromism [34], which is probably due to the intermolecular H bonds formed between the NH_2_ groups and H_2_O, leading to the higher energy of light needed for the excitation and, consequently, the fluorescence spectra shifted to the lower wavelengths for cca. 40–50 nm.

**Figure 1:**
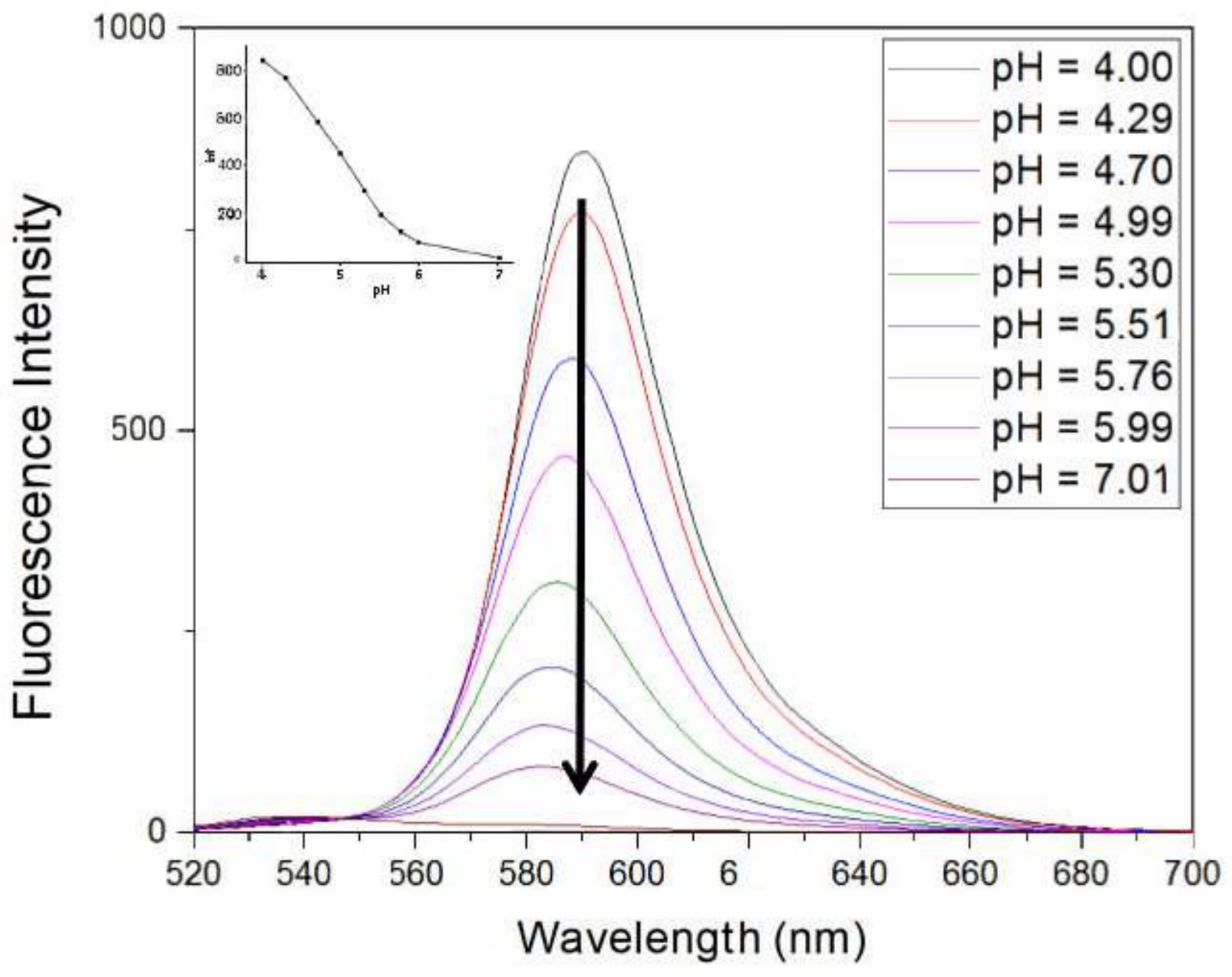
pH response of RhB-EDA in different pH from pH 4 to pH 7 (λ_ex_/λ_em_ = 500 nm/590 nm), and the inset shows the titration curve. Figure 1 shows also double emissions at 538 and 590 nm, but emission at 538 is less pronounced due to the high fluorescence intensity at pH 4. Upon increasing the set-up of instrument parameters like detector voltage and ex/em slits, the double emission at 538 nm is more pronounced (for more information see Section 3.6).

Relatively small changes in pH can sometimes affect the intensity and spectral characteristics of fluorescence RhB-EDA radically. In solution at pH 7.0, it is colourless, but the addition of acid is accompanied in the optical properties via change from transparent to pink. Therefore, the effect of pH on the sensor probe fluorescence characteristic was investigated over a pH range from 4.0 to 7.0 in an MOPS buffer (Figure 1).

**Scheme 2:**
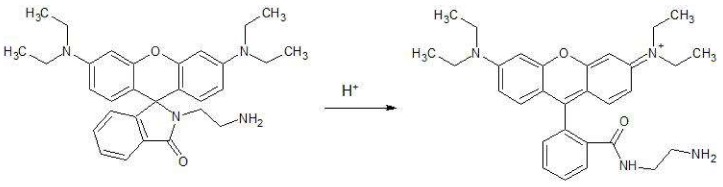
Transformation of spirolactam form of less fluorescent RhB-EDA to highly fluorescent open ring amide in the presence of H^+^ ion [35].

RhB-EDA is a Rhodamine lactam and, as such, is a part of a group of non-fluorescent and colourless compounds marked by intra-molecular spirolactams, which are poised to form highly fluorescent rhodamine derivatives under appropriate conditions via ring opening of the spirolactam [35,36,37]. Upon the addition of H^+^ ions (Scheme 2), its structure undergoes a change from the spirolactam to an open ring amide, resulting in a magenta-coloured, highly fluorescent compound [35,38]. The fluorescence spectra showed that RhB-EDA fluorescence is the highest at pH 4. The fluorescence intensity of RhB-EDA decreases with pH increase. pH > 8 cannot be used for sensing due to the risk of precipitation of insoluble RhB-EDA. Moreover, at pH 4, quenching of the fluorescence in the presence of different concentrations of Ag^+^ was insignificant. However, on the other hand, at pH 7, the response to the different concentration of the Ag^+^ ions was significant. This is probably due to ring open amide structure at pH 4 and spirolactam form at pH 7. Therefore, pH 7 was chosen for further experiments.

It is well known that fluorescence probes can be photo unstable [33]. Therefore, we tested the photostability of the RhB-EDA dye in buffer solution. For this reason, the fluorescence intensity of RhB-EDA was measured in the absence of Ag^+^ ions; it was recorded every 0.5 s over an 80 min period (λ_ex_/_λem_ was selected at 500/538 nm. Slit ex/em 5.0/9.0 nm). The fluorescence signal increased by ∆I_f_ = +9% after the first 3 min and then, in an additional 77 min, decreased slowly for an additional ∆I_f_ = −4%. These effects can also be attributed to the fluctuation of the excitation light source [39].

### 3.3. Response of RhB-EDA Fluorescent Probe to Various Metals

The effect on the fluorescence intensity of an RhB-EDA fluorescent probe in the presence of a range of environmentally and physiologically important metal ions was, furthermore, investigated in a DMSO:H_2_O (1:9) solvent system buffered with MOPS at pH 7.0 with λ_ex_ and λ_em_ at 500/538 nm (Table 1).

The effect of incubation time was investigated to obtain the best sensitivity. After the addition of 10^−4^ M of Ag^+^ ions, the fluorescence of RhB-EDA started to decrease, and, after 20 min, remained constant. Based on that, a 20 min incubation time was chosen to obtain stable and comparable results on Ag^+^ ions’ response (figure not shown).

The selectivity behaviour is obviously one of the most important characteristics of a sensor probe, which reflects the relative response of the probe for primary ion over diverse ions.

**Scheme 3:**
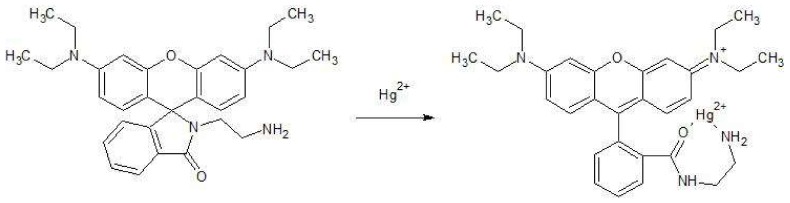
Transformation of spirolactam form of less fluorescent RhB-EDA to a highly fluorescent open ring amide in the presence of Hg^2+^ ions [41].

**Figure 2:**
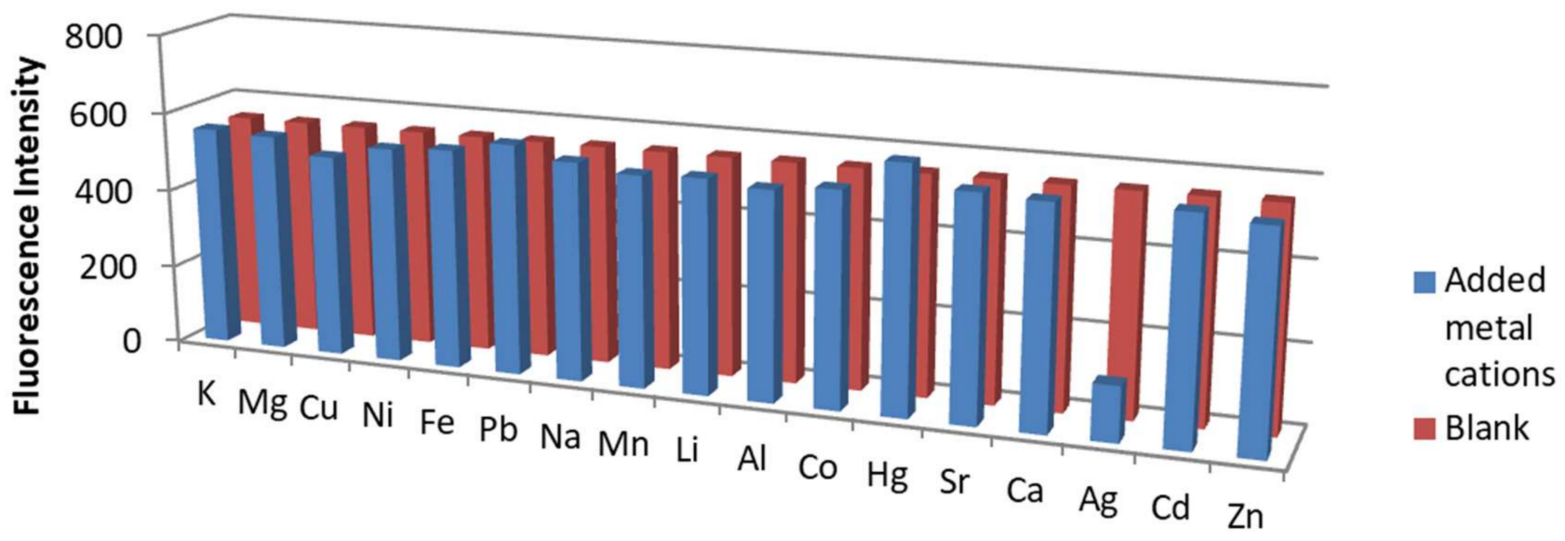
Fluorescence response of *N*-(Rhodamine-B)lactam-ethylenediamine (10^−4^ M) at 538 nm (λ_ex_ = 500 nm) as a function of various added metal cations (10^−3^ M) in aqueous solution of 100 mM phosphate buffer (pH 7). From left to right: K^+^, Mg^2+^, Cu^2+^, Ni^2+^, Fe^2+^, Pb^2+^, Na^+^, Mn^2+^, Li^+^, Al^3+^, Co^2+^, Hg^2+^, Sr^2+^, Ca^2+^, Ag^+^, Cd^2+^, Zn^2+^. λ_ex_/λ_em_ was selected at 500/538 nm. Slit ex/em 5.0/9.0 nm.

**Figure 3:**
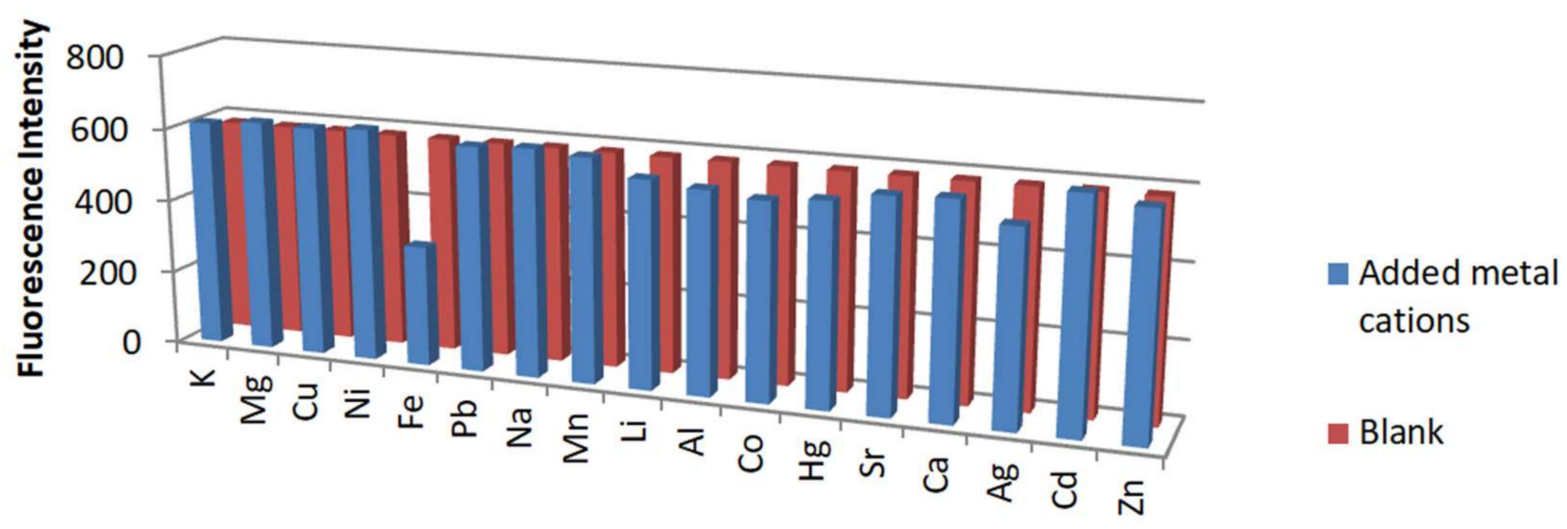
Fluorescence response of Rhodamine B (10^−7^ M) at 575 nm (λ_ex_ = 555 nm) as a function of various added metal cations (10^−3^ M) in aqueous solution of 100 mM phosphate buffer (pH 7). From left to right: K^+^, Mg^2+^, Cu^2+^, Ni^2+^, Fe^2+^, Pb^2+^, Na^+^, Mn^2+^, Li^+^, Al^3+^, Co^2+^, Hg^2+^, Sr^2+^, Ca^2+^, Ag^+^, Cd^2+^, Zn^2+^. λ_ex_/λ_em_ was selected at 555/575 nm. Slit ex/em 6.0/6.0 nm.

The response of the sensor probe RhB-EDA to Ag^+^ ions, as well to other environmentally relevant metal ions, was examined, including K^+^, Mg^2+^, Cu^2+^, Ni^2+^, Fe^2+^, Pb^2+^, Na^+^, Mn^2+^, Li^+^, Al^3+^, Co^2+^, Hg^2+^, Sr^2+^, Ca^2+^, Ag^+^, Cd^2+^ and Zn^2+^. Table 1 and Figure 2 show the signal of the blank sample and change in the fluorescent signal in the presence of different metal ions. Figure 3 also shows that the sensor probe is less sensitive to other metal ions, since the fluorescence signal at 538 nm was significantly decreased (∆I_f_ = −75.05%) only in the presence of Ag^+^ ions. The Ag^+^ sensor probes are usually also sensitive to Hg^+^ ions. This is due to the fact that Ag^+^ and Hg^2+^ are soft ions that display rather similar complex stabilities and, potentially, interfere with each other [40]. In our case, the probe did not behave in such a way. Namely, the fluorescence signal of the probe in the presence of Hg^2+^ ions was even enhanced by about ∆I_f_ = +11.29% (Figure 3). Comparable to the description of H^+^ optical ion behaviour (enhanced fluorescence, Scheme 2), we assume that structure undergoes a change from the spirolactam to an open ring amide and, consequently, carbonyl oxygen and primary amine provide nice binding pockets for Hg^2+^ (enhanced fluorescence, Scheme 3) and also increase the fluorescence intensity [28,36,37,41,42] Ag^+^ ions, on the contrary, show opposite behaviour, and the fluorescence intensity is quenched, probably due to closed spirolactam form and the reduced conjugation of RhB-EDA.

**Figure 4:**
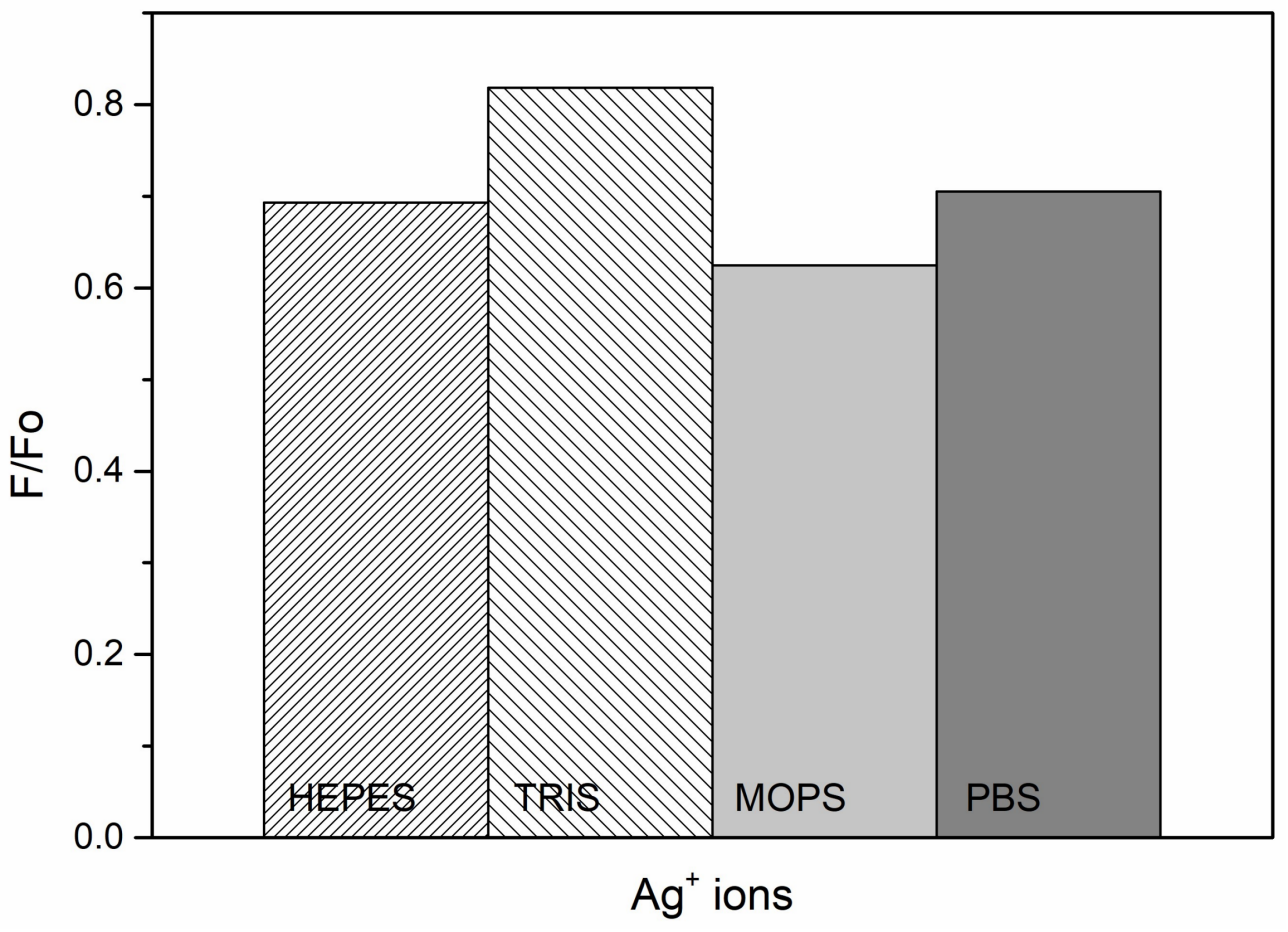
Buffer effect (HEPES, TRIS, MOPS and PBS) on fluorescence intensity of RhB-EDA in the presence of 10^−4^ M Ag^+^ ions. λ_ex_/λ_em_ was selected at 500/538 nm. Slit ex/em 6.0/6.0 nm.

In order to show the impact of the new sensor probe structure RhB-EDA (gained by simple organic modification of RhB), on its sensitivity, we also tested the response of RhB to various metal ions. Figure 4 shows the fluorescence response of RhB itself to various metal ions. The signal change of RhB in the presence of Ag^+^ ions is rather small (∆I_f_ = −5.69%; Table 1 and Figure 3). The fluorescence signal change in the case of RhB-EDA in the presence of Ag^+^ ions is much more significant (∆I_f_ = −75%; Figure 2). The sensor probe RhB showed the biggest sensitivity towards Fe^2+^ ions (∆I_f_ = −41.30%; Figure 3), while RhB-EDA shows almost no response to the Fe^2+^ ions (∆I_f_ = +0.08%; in the range of measurement error area). The sensitivity of RhB towards Fe^2+^ is still smaller than the sensitivity of RhB-EDA to Ag^+^ ions. The selectivity test confirms that the RhB and RhB-EDA sensor probes are different, not only in their spectral properties, but also in their sensor behaviour, due to their different structures. This confirms our idea that, with quite easy synthesis, we can obtain a new sensor probe with different sensor characteristics compared to the RhB probe.

### 3.4. Effect of Buffers

**Figure 5:**
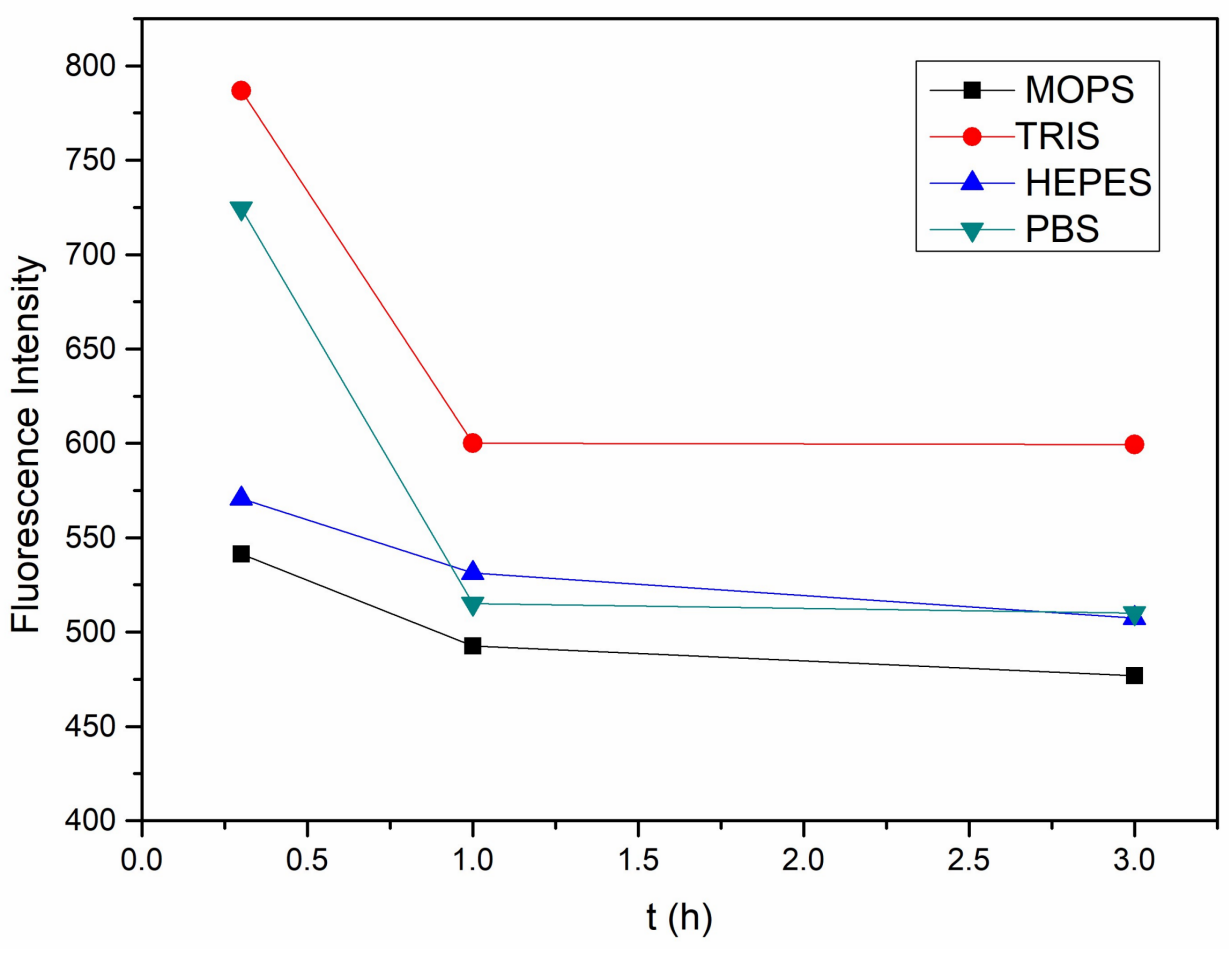
Buffer effect (HEPES, TRIS, MOPS and PBS) on fluorescence intensity of RhB-EDA in the presence of 10^−4^ M Ag^+^ ions depending on time (measurements were taken at time 0, after 60 min and 3 h). λ_ex_/λ_em_ was selected at 500/538 nm. Slit ex/em 6.0/6.0 nm.

In several previous studies, the effect of the buffer on the fluorescence intensity has been demonstrated [43,44]. The effect of buffer type on the RhB-EDA fluorescence response to Ag^+^ ion has been tested. Four different buffers (HEPES, TRIS, MOPS and phosphate buffer) have been used (Figure 4). The concentration of RhB-EDA and Ag^+^ ions in the buffers of pH 7 was 10^−4^ M and 10^−3^ M, respectively. HEPES and phosphate have no significant effect on the sensitivity of RhB-EDA to Ag^+^ ions. On the other hand, the TRIS buffer reduced the sensitivity of RhB-EDA towards Ag^+^ ions by ∆I_f_ = −15%. The highest sensitivity of RhB-EDA has enhanced by ∆I_f_ = +11% when the MOPS buffer has been used. The highest signal stability was also observed in the MOPS buffer (Figure 5). Therefore, further studies were carried out in a MOPS buffer after 20 min.

### 3.5. Effect of Various pHs of RhB-EDA Fluorescence in the Presence of Ag^+^ Ions

**Figure 6:**
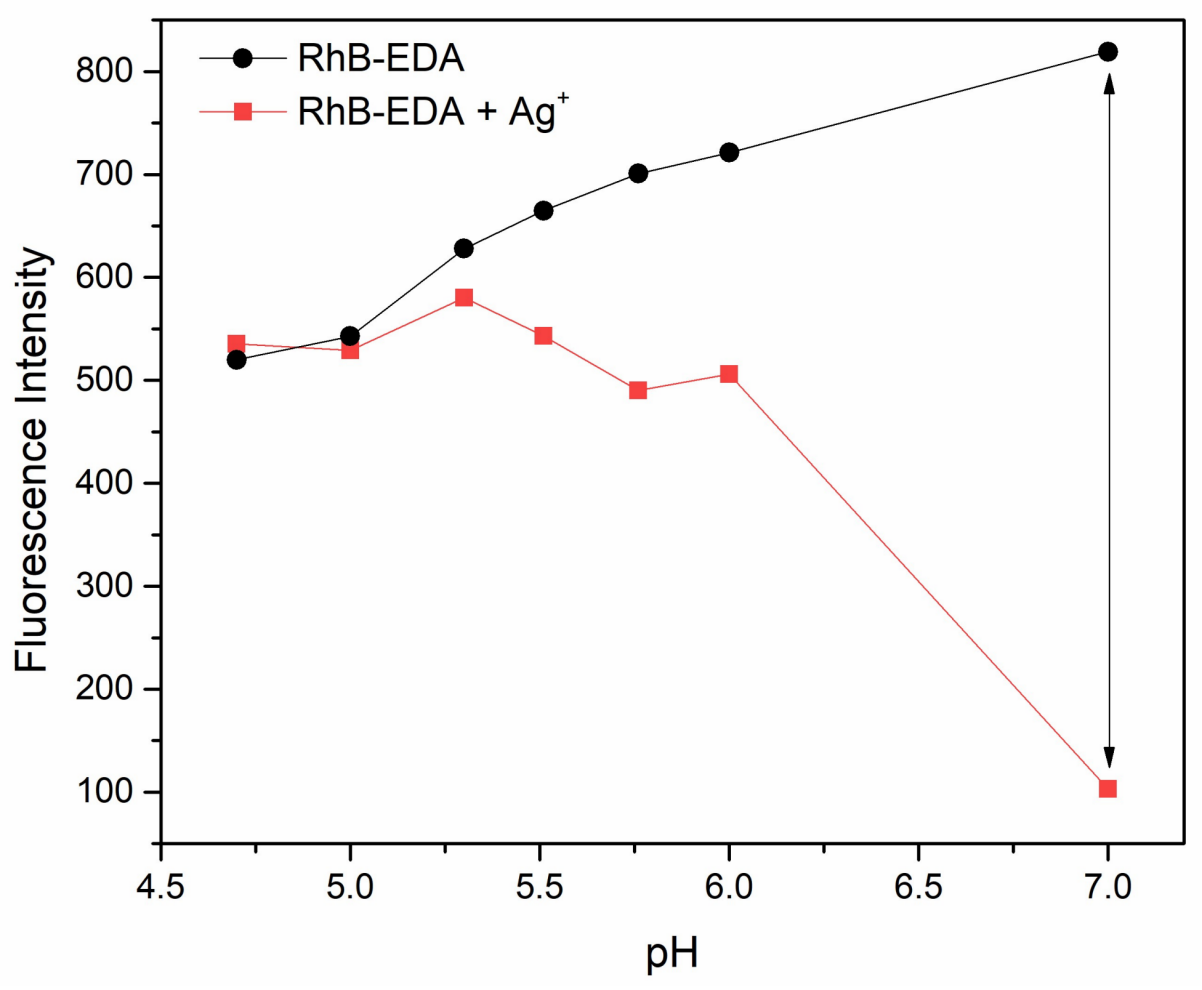
Changes of fluorescence intensity at 538 nm (λ_ex_ = 500 nm) of RhB-EDA (10^−4^ M) in aqueous solution of MOPS buffer measured with and without Ag^+^ (10^−3^ M) as a function of pH.

Many sensor probes that have functional groups, such as –OH, –CO_2_H and –NH, etc., are pH sensitive [45,46]. Therefore, we also tested the pH effect on RhB-EDA change response in the presence of Ag^+^ ions. Figure 6 shows that the RhB-EDA sensor probe is sensitive to an acidic pH effect, and silver ions are known to form silver hydroxide at high pHs. Therefore, the pH effect was tested only in an acid to neutral pH range (pH 4–7). The biggest signal change (∆I_f_ = −700) of RhB-EDA (10^−4^ M) fluorescence intensity was obtained in the presence of Ag^+^ ions (10^−3^ M) at pH 7 (Figure 6).

### 3.6. Sensitivity of RhB-EDA to Ag^+^

**Figure 7:**
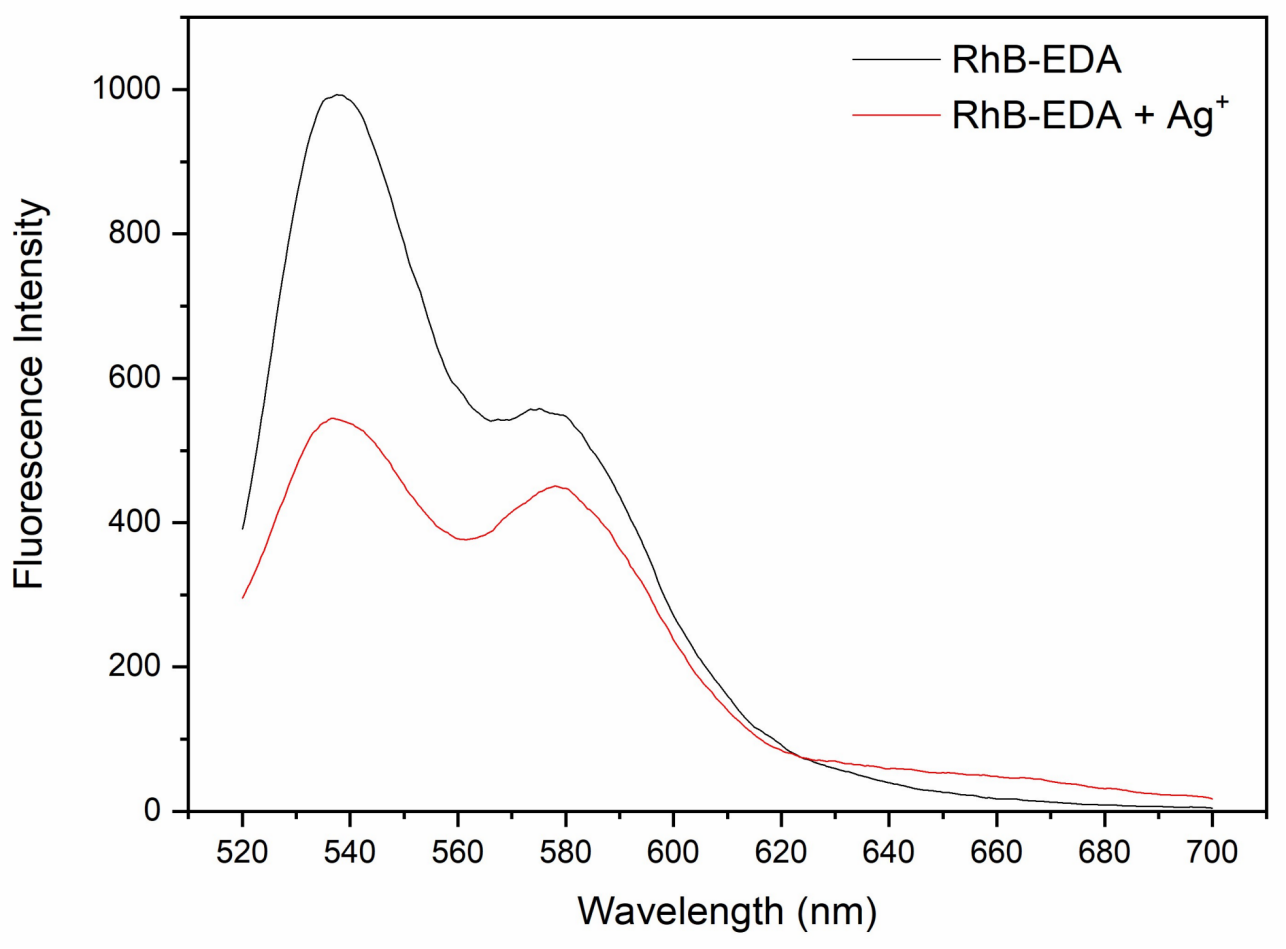
Fluorescence spectra of RhB-EDA alone (10^−4^ M) and RhB-EDA (10^−4^ M) after addition of 10^−4^ M Ag^+^ ions (λ_ex_/λ_em_ = 500/538 nm).

Figure 7 shows the fluorescence spectra of the RhB-EDA probe in the absence, and in the presence, of Ag^+^ ions (10^−4^ M). In the absence of Ag^+^ ions, RhB-EDA exhibits two emission peaks at 538 nm and 590 nm. Since the sensitivity of RhB-EDA probe towards Ag^+^ ions was higher at 538 nm than at 590 nm, the emission peak at 538 nm was chosen for further investigation.

The presence of Ag^+^ ions results in significant fluorescence quenching (∆I_f_ = −75.05%) after 20 min incubation in an MOPS buffer. For the sensitivity study (Figure 5), different concentrations of Ag^+^ ions in the range from 0.43 × 10^−3^ to 10^−6^ M were investigated in the presence of an RhB-EDA sensor probe. The fluorescence intensity of the RhB-EDA sensor probe decreases with the increase of Ag^+^ concentration. Figure 6 shows the value of F/F_0_ plotted against the concentration of Ag^+^ ions, where F_0_ and F are the fluorescence intensity of RhB-EDA, without and with Ag^+^, respectively. No further decrease in the fluorescence intensity was observed after µM concentration range of Ag^+^ ions range (>10^−6^ M) were used, indicating that the interaction between RhB-EDA and Ag^+^ reached the balance.

**Figure 8:**
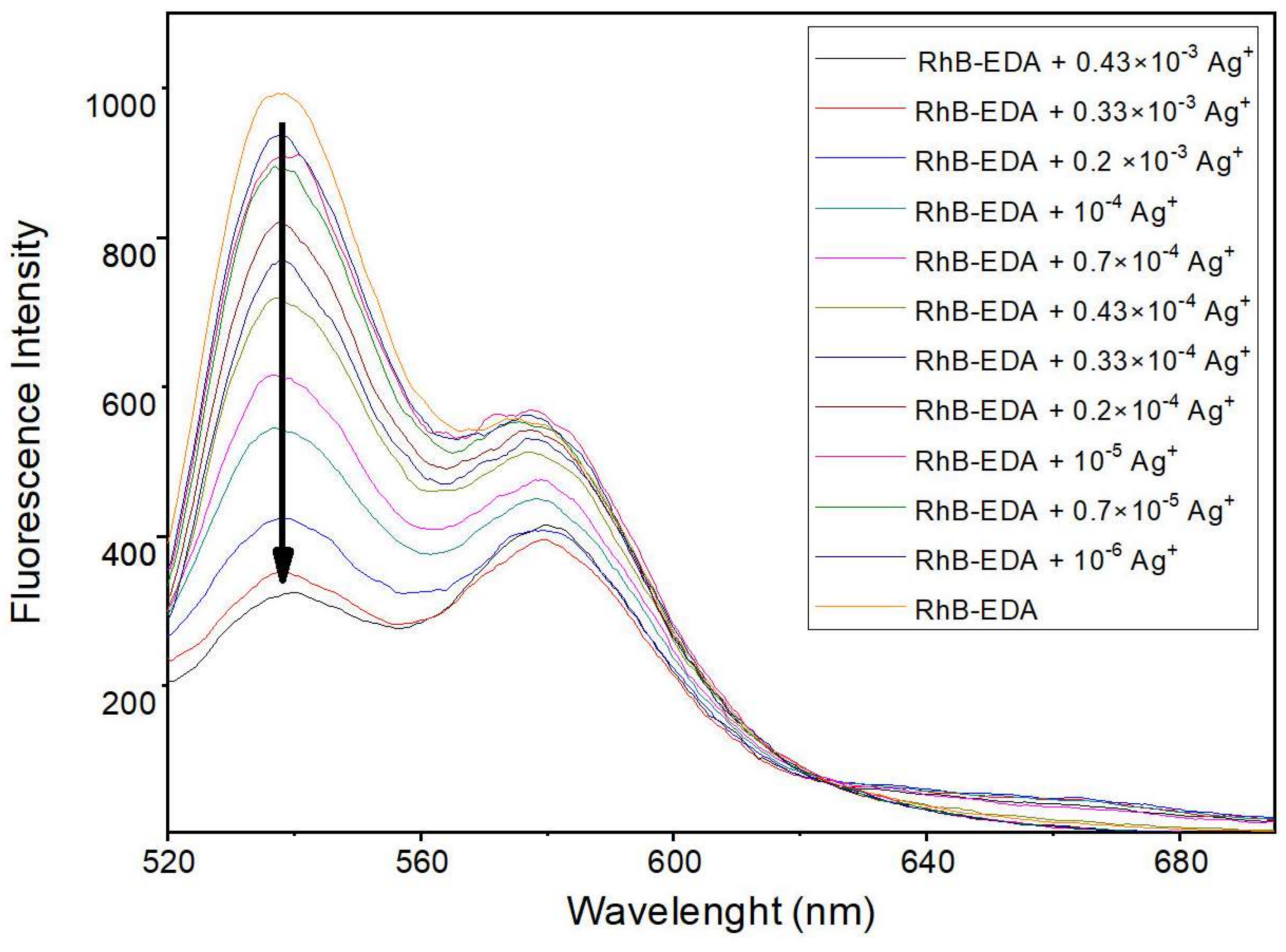
Fluorescence spectra of 100 µM RHB-EDA probe in the presence of various concentrations of Ag^+^ ions at pH 7 (DMSO:MOPS:H_2_O = 3:14:13) (λ_ex_/λ_em_ = 500/538 nm).

**Figure 9:**
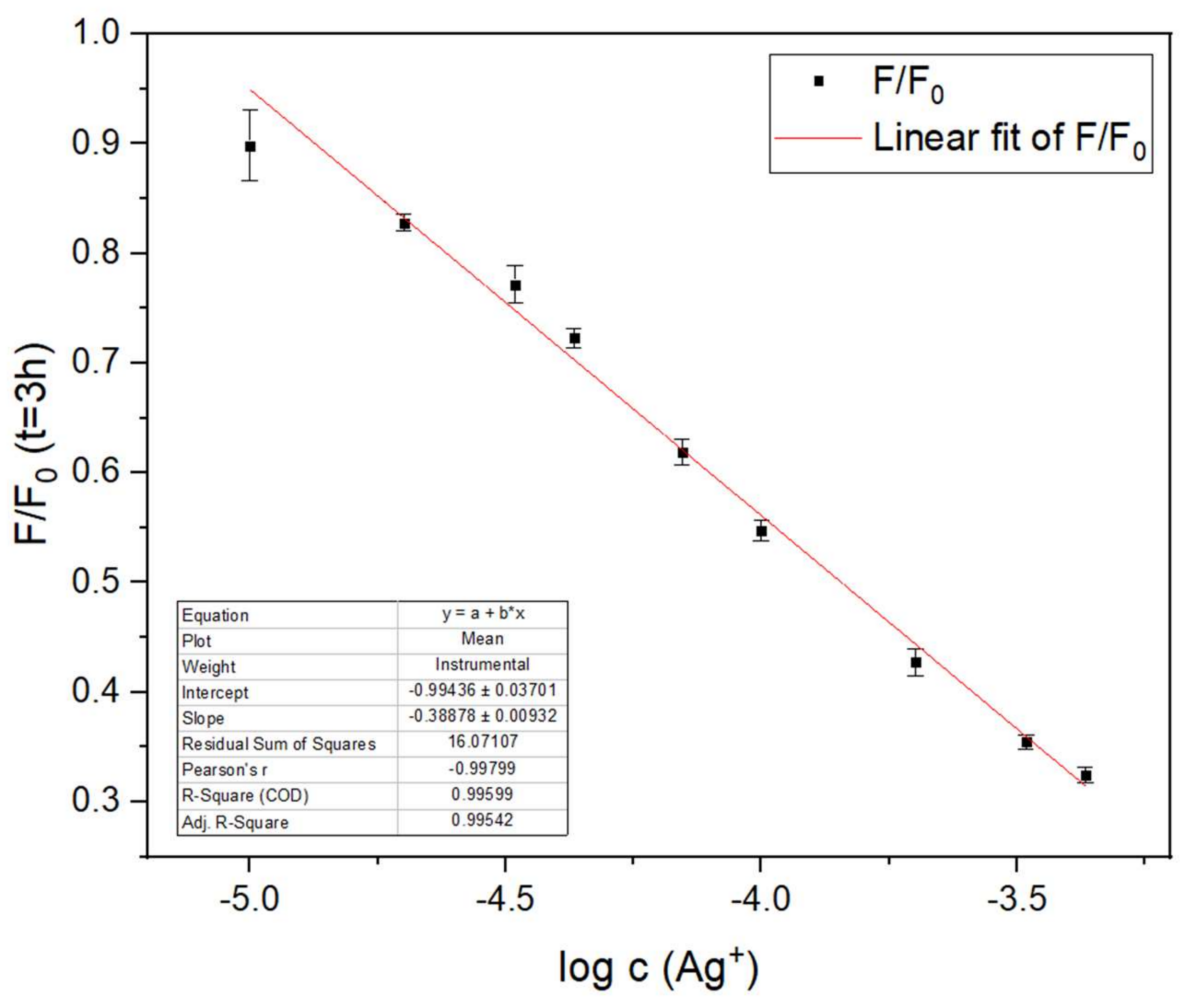
Calibration plot of RhB-EDA in the presence of various Ag^+^ ions concentrations (0.43 × 10^−3^, 0.33 × 10^−3^, 0.2 × 10^−3^, 10^−4^, 0.7 × 10^−4^, 0.43 × 10^−4^, 0.33 × 10^−4^, 0.2 × 10^−4^, 10^−5^, 0.7 × 10^−5^, 10^−6^ M) in MOPS buffer at pH 7 (λ_ex_/λ_em_ = 500/538 nm).

Figure 8 shows the presented decrease of fluorescent intensity of the RhB-EDA probe in the presence of various concentrations of Ag^+^ ions. Figure 9 presents a calibration plot of RhB-EDA in the presence of various concentrations of Ag^+^ ions. The limit of detection is 0.1 µM of Ag^+^ (LOD, defined as three times the standard deviation of blank).

### 3.7. Comparison with Developed Probe-Based Sensors Intended for on-Site Detection of Ag^+^

In Table 2, the comparison data of developed probes for the on-site determination of the Ag^+^ are listed, along with the synthesized RhB-EDA analyzed within this work. 

One can see that the use of fluorescence-based technique is prevailing. H_2_L∙2Br [11] was compared with a lower amount of interference, and determination of Ag^+^ is done in 100% organic solution; therefore, this fluorescent chemodosimeter is not a candidate for measuring Ag^+^ ions in real-life applications. Since 100% organic solvent is used for detection of Ag^+^, 2-Pyridine-*1H*-benzo[*d*]imidazole based conjugated polymer [15] and bis(isatin hydrazonyl) calix[4]arene based dual receptor [18] are also inappropriate probes for real-time application. A phenazine derivate [17] in addition to using a partially organic solvent (DMSO/H_2_O (6:4)), this probe was not tested on Hg^2+^ as interference, though it is well known that Ag^+^ sensor probes are usually also sensitive to Hg^+^ ions [40]. Apart from phenazine derivate, S-GQDs [13] were also not tested in the presence of Hg^2+^ ions, so selectivity of this probe is also questionable. No selectivity, due to a similar response to Ag^+^ and Hg^2+^, showed a Squaraine based probe [22]. Fe_3_O_4_@Au [21] has interference in the presence of a high concentration of Hg^2+^ ions, and measurement was taken in media with high pH (pH > 12), which is less suitable for real-time samples. In the case of an FQ-crown [19], all tested ions (K^+^, Mg^2+^, Cu^2+^, Ni^2+^, Fe^3+^, Pb^2+^, Mn^2+^, Co^2+^, Hg^2+^, Cr^3+^, Ca^2 +^, Cd^2+^, Zn^2+^) slightly interfere with Ag^+^. Fluorescent N doped C-dots [14] have quite a similar response to Hg^2+^ and Ag^+^, and the impact of different pH is not known. Another Rhodamine B derivate [12] was used as a probe-based sensor with good sensitivity to Ag^+^ but may not be reversible. A change in color results upon binding Ag^+^ to the iodide in an elimination reaction.

The detection limit of the proposed RhB-EDA probe is lower compared to probes with similar detection mechanisms [12,17,22]. The sensors’ probes [13,14,16] that use nanoparticles have lower limit of detection than the proposed RhB-EDA probe.

Compared to current probes for determination of Ag^+^, the RhB-EDA can be synthesized easily without any complicated processes and used for determination of Ag^+^ in aqueous media. Moreover, as the probe displays a selective fluorescence response at wavelength 538 nm, it is more suitable for practical determination due to compatibility with cheap light sources.

## 4. Conclusions

We can conclude that we have synthesised and optimised a fluorescent sensor probe successfully, based on organic modification of a well-known RhB fluorescent probe. As expected, the new probe showed significant changes in its behaviour as an optical sensor probe. By this modification, we obtained a highly sensitive and selective probe for sensing Ag^+^ ions. The Ag^+^ sensor probes are usually also sensitive to Hg^2+^ ions, but RhB-EDA shows only selective optical sensitivities towards Ag^+^ ions. Furthermore, it was found that the probe response is also influenced by the type of buffer. Among all those tested, it was shown that the sensitivity toward Ag^+^ ions is the highest in the MOPS buffer. The linear range for Ag^+^ ions’ detection is from 0.43 × 10^−3^ to 10^−6^ M and LOD is 0.1 µM. Since all sensing characteristics of the RhB-EDA were tested in green aqueous media and not in different organic solvent solutions, this probe may be a good candidate for measuring Ag^+^ ions in real-life applications. We show successfully that simple, fast and inexpensive synthesis can contribute to the novel fluorescent Ag^+^ sensor probe.

Moreover, our next step will be to immobilise this sensor probe in sol-gel thin film and in SiO_2_ nanoparticles, since, in our previous work, we have shown that this can improve the sensor characteristics. Therefore, the goal of the research was to synthesise a sensor probe for Ag^+^ ion detection that would be stable, relatively inexpensive, easy to produce and useful in green aqueous media.

## Figures and Tables

**Table 1:** Fluorescence signal change of RhB and RhB-EDA to various metals ions at pH 7.

	K^+^	Mg^2+^	Cu^2+^	Ni^2+^	Fe^2+^	Pb^2+^	Na^+^	Mn^2+^	Li^+^	Al^3+^	Co^2+^	Hg^2+^	Sr^2+^	Ca^2+^	Ag^+^	Cd^2+^	Zn^2+^
∆I_f_ (RhB-EDA) (%)	0.51	−0.61	−7.69	−1.68	+0.08	+4.96	−0.56	−3.82	−2.75	−5.21	−2.59	+11.29	+1.26	−0.16	−75.05	+0.53	−2.21
∆I_f_ (RhB) (%)	+11.07	+13.32	+12.57	+14.05	−41.30	+9.96	+11.30	+9.35	+1.13	−1.31	−3.99	−1.60	+3.06	+4.10	−5.69	+11.43	+7.36

**Table 2:** Comparison data of developed probe-based sensors intended for on-site detection of Ag^+.^

Indicator	Tested Interference	Solutions	LOD	Remark	[Ref.]
RhB-EDA	K^+^, Mg^2+^, Cu^2+^, Ni^2+^, Fe^2+^, Pb^2+^, Na^+^, Mn^2+^, Li^+^, Al^3+^, Co^2+^, Hg^2+^, Sr^2+^, Ca^2+^, Cd^2+^, Zn^2+^		0.1 µM		our work
H_2_L∙2Br	Cu^2+^, Ni^2+^, Fe^2+^, Pb^2+^, Mn^2+^, Co^2+^, Hg^2+^, Cr^3+^, Zn^2+^	Acetonitrile/dichloromethane (1:1)	Not given	F, Low number of tested interference, 100% organic solution	[11]
Rhodamine B derivate	Mg^2+^, Cu^+^, Cu^2+^, Ba^2+^, Ni^2+^, Fe^3+^, Pd^2+^, Mn^2+^, Co^2+^, Hg^2+^, Hg^+^, Ca^2+^, Cd^2+^, Cr^3+^, Zn^2+^	20% ethanolic water	0.144 µM	F, Rhodamine B derivate, less number of tested interference, it is not reversible	[12]
S-GQDs	K^+^, Mg^2+^, Cu^2+^, Ni^2+^, Fe^2+^, Pb^2+^, Na^+^, Al^3+^, Co^2+^, Ca^2+^, Cd^2+^ Zn^2+^	HEPES buffer solution	30 nM	F, Not tested on Hg^+^ as well know interference for Ag^+^ ot tested	[13]
NCDs	Mg^2+^, Cu^2+^, Ni^2+^, Fe^2+^, Fe^3+^, Ba^2+^, Mn^2+^, Pb^2+^, Cr^6+^, Cr^3+^, Hg^2+^, Ca^2+^, Cd^2+^, Zn^2+^	100% water solution	1 nM	F, No selectivity: same response to Ag^+^ and Hg^2+^, not tested to different pH	[14]
Polymer (ethyl 2-(2-(pyridin-2-yl)-1*H*-benzo[*d*]imidazol-1-yl) acetate units)	Ba^2+^, Mg^2+^, Cr^3+^, Ni^2+^, Fe^2+^, Na^+^, Mn^2+^, Li^+^, Al^3+^, Co^3+^, Hg^2+^, Sn^2+^, Ca^2+^, Cd^2+^, Zn^2+^	THF solution	Not given	F, 100% organic solution	[15]
MnO_2_ nanosheets + ligand-DNA	Ba^2+^, Mg^2+^, Fe^3+^, Mn^2+^, Li^+^, Al^3+^, Cu^2+^, Hg^2+^, Ca^2+^, Cd^2+^, Zn^2+^	Ammonium acetate-buffered saline solution	9.1 nM		[16]
Phenazine derivate	Fe^3^+, Hg^2+^, Ca^2+^, Cu^2+^, Co^2+^, Ni^2+^, Cd^2+^, Pb^2+^, Zn^2+^, Cr^3+^, Mg^2+^	DMSO/H_2_O (6:4) HEPES buffer (pH = 7.2)	0.325 µM	A, F, Long and complicated synthesis	[17]
bis(isatin hydrazonyl) calix[4]arene based dual receptors	Cu^2+^, Ni^2+^, Fe^2+^, Mn^2+^, Co^2+^, Hg^2+^, Cd^2+^, Zn^2+^	THF solution	3.98 µM	A, 100% organic solution	[18]
FQ-crown	K^+^, Mg^2+^, Cu^2+^, Ni^2+^, Fe^3+^, Pb^2+^, Mn^2+^, Co^2+^, Hg^2+^, Cr^3+^, Ca^2 +^, Cd^2+^, Zn^2+^	EtOH	Not given	F, All tested interference slightly interfere	[19]
DNA probe	Cu^2+^, Hg^2+^, Ca^2+^, Al^3+^, Na^+^, K^+^, Pb^2+^, Cd^2+^, Zn^2+^	10 mM Tris-HCl with 50 mM [Ru(NH_3_)_6_]^3+^ (pH 7.4)	0.1 nM	E, complicated preparation of probe	[20]
Fe_3_O_4_@Au	Mg^2+^, Cu^2+^, Fe^3+^, Co^2+^, Hg^2+^, Ca^2+^, Al^3+^, Pb^2+^, Cd^2+^, Zn^2+^	Water, pH > 12	59 nM	E, interference with high concentration of Hg^2+^, measuring in media with high pH	[21]
Squaraine dye	K^+^, Mg^2+^, Cu^2+^, Ni^2+^, Fe^3+^, Pb^2+^, Na^+^, Mn^2+^, Li^+^, Al^3+^, Co^2+^, Hg^2+^, Ca^2+^, Cd^2+^, Ba^2+^, Zn^2+^	EtOH/H_2_0 (50:50, *v*/*v*)	40 µM	F, Long and complicated synthesis, No selectivity: similar response to Ag^+^ and Hg^2+^	[22]

E—electrochemical-based assay; A—absorbance-based assay, F—fluorescence-based assay.
