# Correction: Soršak et al. Design and Investigation of Optical Properties of *N*-(Rhodamine-B)-Lactam-Ethylenediamine (RhB-EDA) Fluorescent Probe. *Sensors* 2018, *18*, 1201

**DOI:** 10.3390/s24103043

**Published:** 2024-05-11

**Authors:** Eva Soršak, Julija Volmajer Valh, Špela Korent Urek, Aleksandra Lobnik

**Affiliations:** 1 Institute of Engineering Materials and Design, Faculty of Mechanical Engineering, University of Maribor, Smetanova 17, 2000 Maribor, Slovenia; sorsak.eva@gmail.com (E.S.);; 2 Faculty of Chemistry and Chemical Technology, University of Ljubljana, Večna pot 113, 1000 Ljubljana, Slovenia; 3 Institute for Environmental Protection and Sensors, Beloruska 7, 2000 Maribor, Slovenia

## Additional Affiliation(s)

In the published publication [1], there was an error regarding the affiliation**(s)** for **“Eva Soršak”**. In addition to affiliation(s) **“1”**, the updated affiliation(s) should include: **“Faculty of Chemistry and Chemical Technology, University of Ljubljana, Večna pot 113, 1000 Ljubljana, Slovenia”**. The authors state that the scientific conclusions are unaffected. This correction was approved by the Academic Editor. The original publication has also been updated.
